# SOX2 Expression and Transcriptional Activity Identifies a Subpopulation of Cancer Stem Cells in Sarcoma with Prognostic Implications

**DOI:** 10.3390/cancers12040964

**Published:** 2020-04-13

**Authors:** Sofia T. Menendez, Veronica Rey, Lucia Martinez-Cruzado, M. Victoria Gonzalez, Alvaro Morales-Molina, Laura Santos, Verónica Blanco, Carlos Alvarez, Oscar Estupiñan, Eva Allonca, Juan Pablo Rodrigo, Javier García-Castro, Juana Maria Garcia-Pedrero, Rene Rodriguez

**Affiliations:** 1 Instituto de Investigación Sanitaria del Principado de Asturias (ISPA)—Hospital Universitario Central de Asturias, 33011 Oviedo, Spain; 2 Instituto Universitario de Oncología del Principado de Asturias, 33006 Oviedo, Spain; 3 CIBER en oncología (CIBERONC), 28029 Madrid, Spain; 4 Departamento de Cirugía, Universidad de Oviedo, 33006 Oviedo, Spain; 5 Cellular Biotechnology Unit, Instituto de Salud Carlos III, Majadahonda, 28220 Madrid, Spain; 6 Servicio de Anatomía Patológica of the Hospital Universitario Central de Asturias, 33011 Oviedo, Spain; 7 Servicio de Oncología Médica of the Hospital Universitario Central de Asturias, 33011 Oviedo, Spain

**Keywords:** sarcoma, undifferentiated pleomorphic sarcoma, chondrosarcoma, SOX2, cancer stem cells, EC-8042

## Abstract

Stemness in sarcomas is coordinated by the expression of pluripotency factors, like SOX2, in cancer stem cells (CSC). The role of SOX2 in tumor initiation and progression has been well characterized in osteosarcoma. However, the pro-tumorigenic features of SOX2 have been scarcely investigated in other sarcoma subtypes. Here, we show that SOX2 depletion dramatically reduced the ability of undifferentiated pleomorphic sarcoma (UPS) cells to form tumorspheres and to initiate tumor growth. Conversely, SOX2 overexpression resulted in increased in vivo tumorigenicity. Moreover, using a reporter system (SORE6) which allows to monitor viable cells expressing SOX2 and/or OCT4, we found that SORE6+ cells were significantly more tumorigenic than the SORE6- subpopulation. In agreement with this findings, SOX2 expression in sarcoma patients was associated to tumor grade, differentiation, invasive potential and lower patient survival. Finally, we studied the effect of a panel of anti-tumor drugs on the SORE6+ cells of the UPS model and patient-derived chondrosarcoma lines. We found that the mithramycin analogue EC-8042 was the most efficient in reducing SORE6+ cells in vitro and in vivo. Overall, this study demonstrates that SOX2 is a pro-tumorigenic factor with prognostic potential in sarcoma. Moreover, SORE6 transcriptional activity is a bona fide CSC marker in sarcoma and constitutes an excellent biomarker for evaluating the efficacy of anti-tumor treatments on CSC subpopulations.

## 1. Introduction

Similar to normal tissues, the cancer stem cell model proposes that tumors are hierarchically organized and at the apex of this structure there are cells presenting stem cell like properties (cancer stem cells, CSCs) able to self-renew and to differentiate and give rise to the rest of subpopulations present in the tumor [1]. Bona-fide CSCs are those subpopulations within the tumor with capacity to re-initiate tumor growth. Besides, they have enhanced ability to migrate and invade tissues and show increased resistance to chemotherapeutic drugs. A common characteristic of CSC subpopulations is the overexpression of transcription factors responsible for maintenance of the stem cell phenotype in embryonic and adult stem cells like SOX2 (Sex-determining region Y-box protein 2) or OCT4 (POU5F1, POU Class 5 Homeobox 1) [2]. Subpopulations expressing these pluripotency factors have been correlated with tumor progression, drug resistance and the presence of hierarchically organized CSCs in several types of tumors [3,4,5,6,7,8]. In sarcomas, SOX2 has been found overexpressed in CSCs from different subtypes [9,10,11,12,13,14,15,16,17,18] and was described to play specific pro-tumorigenic roles in osteosarcoma [19,20,21]. In addition, OCT-4 expression was also associated to CSCs subpopulations in Ewing sarcoma and osteosarcoma [22,23].

To unequivocally confirm a given factor as a marker for CSCs, the subpopulation of tumor cells expressing it and/or presenting its associated activity must be isolated and demonstrate a higher tumorigenicity in vivo than other subpopulations. Since pluripotency factors are intracellular molecules, the isolation of viable cells expressing these factors cannot be directly achieved using antibody-based flow cytometry. As an alternative method, the use of reporter systems where the expression of a fluorescent protein is driven by the SOX2 and/or OCT4 promoter or by SOX2/OCT4 response elements has proved the tumor-propagating potential of cells expressing pluripotency factors in several tumor models [4,8,22,24,25,26,27,28,29,30,31]. Notably, this strategy allows the real-time tracking of CSCs and the study of their response to anti-tumor treatments or changes in tumor microenvironment.

In a previous work, we developed cell-of-origin models of sarcoma based in the tumor transformation of human mesenchymal stem/stromal cells (MSCs) using relevant oncogenic events [32,33,34]. We found that self-renewed tumorspheres formed by these cells showed increased expression of several CSC-related genes, including SOX2. Furthermore, by comparing the tumorigenic properties of these models with those of their xenograft-derived cell lines, we found that SOX2 expression was progressively enhanced in CSC-enriched tumorspheres during sarcoma progression toward more aggressive phenotypes, hence highlighting its potential applicability as CSC marker in sarcomas [35]. To further and significantly extend these data, herein we introduced a reporter system to monitor the transcriptional activity due to SOX2 and/or OCT4 (SORE6) [29] in a model of undifferentiated pleomorphic sarcoma (UPS) and chondrosarcoma patient-derived cell lines thus analyzing for the first time the ability of isolated SOX2/OCT4-positive cells as tumor-promoting CSCs in sarcoma. The results of this approach, together with those obtained by SOX2 knockdown and overexpression, indicate that SOX2 expression/activity is a bona fide CSC marker in sarcoma. In addition, this reporter system constitutes an excellent approach for testing the effectiveness and mode of action of anti-tumor drugs on CSC subpopulations.

## 2. Results

### 2.1. SOX2 Expression in Sarcoma Tissue Specimens is Associated to Poor Prognosis and Survival

**Figure 1:**
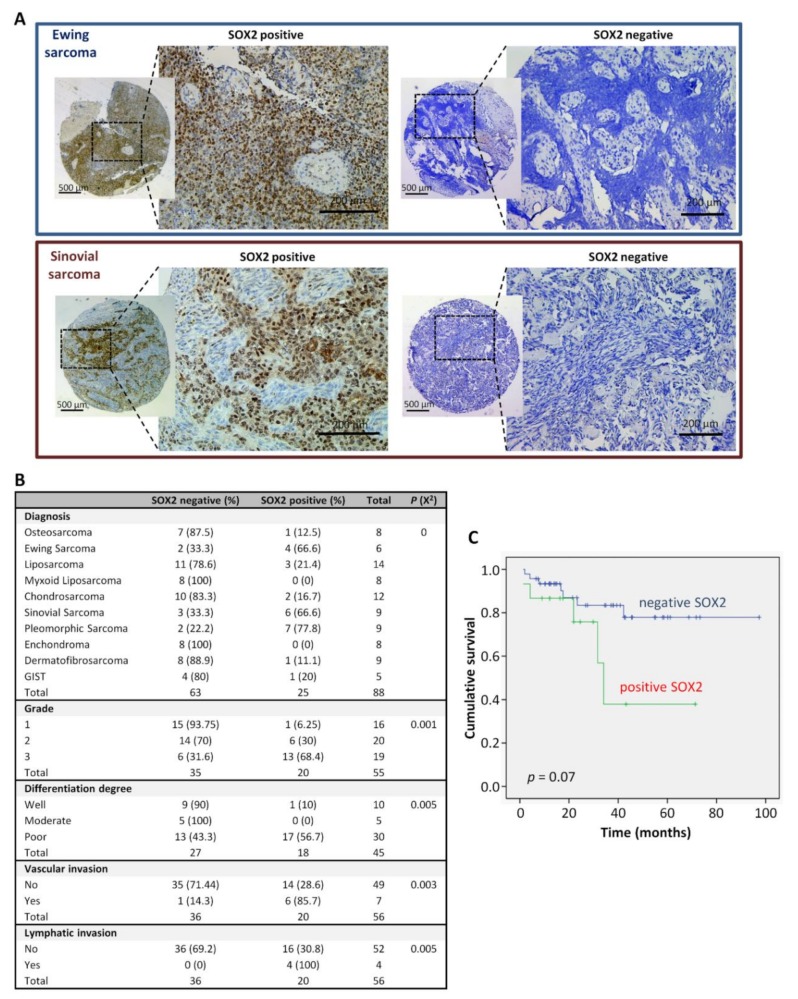
Immunohistochemical analysis of SOX2 expression in sarcoma patients and associations with clinical data. (**A**) Representative examples of the indicated types of sarcoma showing positive or negative SOX2 staining. Scale bars: 200 or 500 μm (insets). (**B**) Distribution of sarcoma cases (N = 88) according to their SOX2 expression level across categories of the indicated patient characteristics and tumor clinicopathologic parameters. *p* values are shown. (**C**) Kaplan-Meier cumulative survival curves categorized by SOX2 protein expression in the cohort of sarcoma patients. *p*-values were estimated using the log-rank test.

We aimed to investigate whether the expression of pluripotency factors such as SOX2 and OCT4 in sarcoma patients is clinically relevant. SOX2 and OCT4 expression was analyzed by immunohistochemistry in a collection of tissue microarrays, including samples from 10 types of sarcomas. Nuclear SOX2 expression was detected in 25 (28.4%) sarcoma samples (Figure 1A,B).

Importantly, SOX2 expression significantly correlated with higher tumor grade (*p* = 0.001), poor differentiation (*p* = 0.005), and the presence of vascular (*p* = 0.003) or lymphatic invasion (*p* = 0.005) (Figure 1B). Moreover, SOX2-negative cases showed a trend for a longer survival time when compared to those expressing this factor (80 months (CI 69-92) vs 42 months (CI 25-59), respectively; HR 2,8; *p* = 0.07). The 5-year survival rate was 78% for negative cases and 38% for positive cases (Figure 1C).

On the other hand, nuclear expression of OCT4 was only detected in 10 cases (11%) and all of them displayed weak staining (Figure S1A,B). We did not find any significant association between OCT4 expression and clinical parameters. However, a strong correlation between SOX2 and OCT4 expression was observed, all OCT4-positive cases were also positive for SOX2 expression (Figure S1B).

In summary, we found that SOX2, but not OCT4, correlated with advanced tumor stages, aggressive phenotypes and poor prognosis in sarcoma patients. According to these data SOX2, rather than OCT4, might primarily play an active role in the initiation and progression of sarcomas.

### 2.2. SOX2 Is Required to Maintain the Tumorigenic Potential in Sarcoma Cells

**Figure 2:**
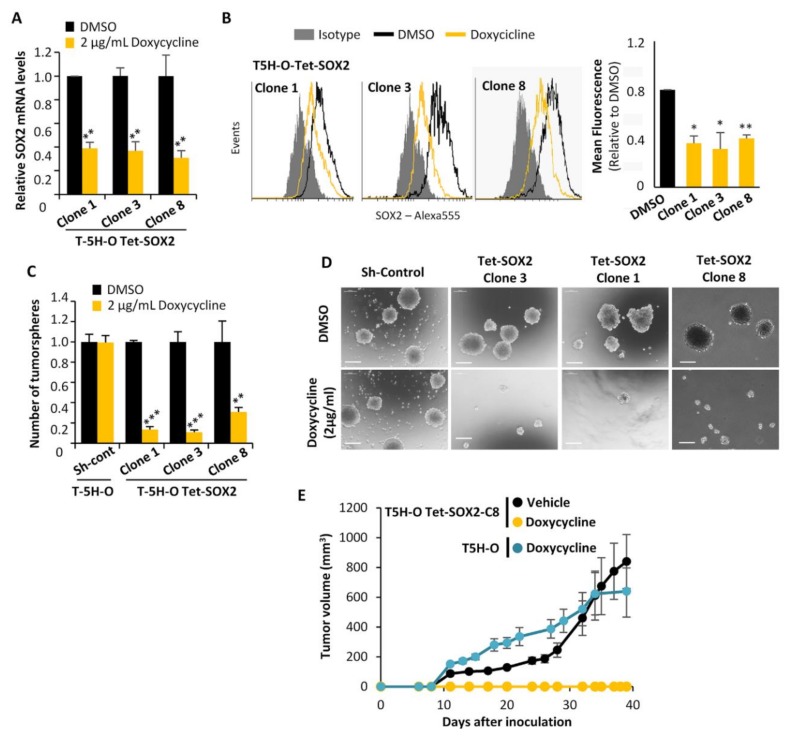
The depletion of SOX2 strongly reduced the tumorigenic potential of sarcoma cells. (**A**,**B**) Relative mRNA expression (**A**) and flow cytometry analysis (**B**) of SOX2 in three clones of T-5H-O cells carrying a doxycycline-inducible SOX2 shRNA in the presence or absence of 2 μg/mL doxycycline for 48 h. (**C**,**D**) Average number of three independent experiments (**C**) and representative images (**D**) of tumorspheres formed by the parental T-5H-O cells transduced with control ShRNA or the T-5H-O clones carrying a doxycycline-inducible SOX2 shRNA in the presence or absence of 2 μg/mL doxycycline for 48 h. Scale bars = 200 μm. (**E**) T-5H-O and T-5H-O-Tet-SOX2-C8 cells were pretreated as indicated with vehicle (DMSO) or 2 μg/mL doxycycline for 72 h prior to the subcutaneous inoculation of 1 × 10^4^ cells in immunodeficient mice (*n* = 7 mice per series). Upon inoculation mice were treated daily with vehicle (saline) or doxycycline (50 mg/kg) via intraperitoneal. Tumor growth (mean volume) kinetics of each series are represented. Error bars represent the standard deviation and asterisks indicate statistically significant differences with control groups (*: *p* < 0.05, **: *p* < 0.005, ***: *p* < 0.0005; two-sided Student *t* test).

To study the possible pro-tumorigenic role of SOX2 in sarcoma, we performed knockdown experiments in T-5H-O cells, a previously described cell-of-origin model of UPS [32,33,34]. First, we transduced T-5H-O cells with lentiviral particles carrying a doxycycline-inducible SOX2 shRNA and selected three clones (T-5H-O-Tet-shSOX2#1, #3 and #8) that showed efficient depletion of SOX2 expression upon doxycycline treatment (Figure 2A,B). According to the reciprocal regulation of these pluripotency factors [2], SOX2-depleted cells also displayed reduced expression of OCT4 (Figure S2). Consistent with the role of SOX2 in stemness, its depletion in all the clones significantly decreased tumorsphere formation (Figure 2C,D). More importantly, doxycycline treatment of mice inoculated with doxycycline-pretreated T-5H-O-Tet-shSOX2#8 cells, but not with parental T-5H-O cells, was sufficient to prevent in vivo tumor growth (Figure 2E). In line with these results, we found a significant reduction in both the ability to form colonies in soft-agar, a surrogate in vitro transformation assay, and the capacity to grow as tumorspheres upon depletion of SOX2 expression in T5H-O cells using another, non-conditional, shRNA (Figure S3A–E) or a siRNA (Figure S3F–J).

**Figure 3:**
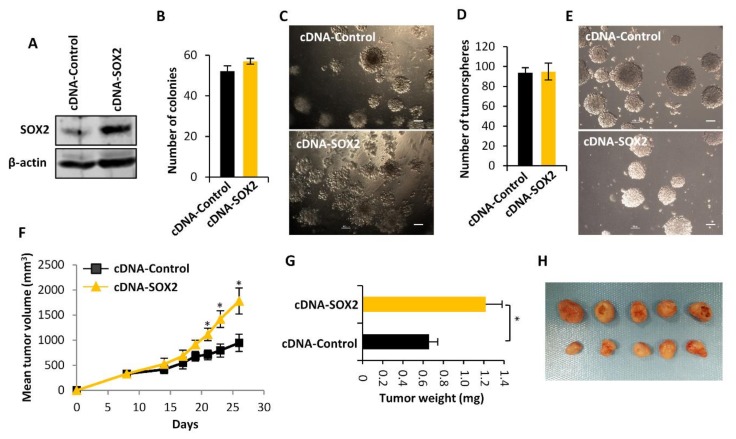
The overexpression of SOX2 increased the tumorigenic potential of sarcoma cells. T-5H-0 cells were stably transduced using lentiviral vector to express EGFP cDNA (cDNA Control) or human SOX2 cDNA (cDNA SOX2) sequences. (**A**) Western blotting analysis of SOX2 expression. (**B**,**C**) Quantification of the number of colonies formed (*n* = 3 independent experiments) (**B**) and representative images of soft agar assays (**C**) performed with control and SOX2-overexpressing cells. (**D**,**E**) Average number of tumorspheres generated in three independent experiments (**D**) and representative images of the indicated tumorsphere cultures (**E**). Scale bars = 100 μm. (**F**–**H**) Evaluation of tumor growth observed after the inoculation of 1 × 10^6^ cells stably transfected with cDNA Control or cDNA SOX2 in immunodefficient mice. Tumor growth kinetics (**F**), tumor weights (**G**) and images of tumors at the end of the experiment (**H**) are presented. Error bars represent the standard deviation and asterisks indicate statistically significant differences (*: *p* < 0.05 by two-sided Student *t* test).

To further confirm the SOX2-driven tumorigenic properties in sarcoma cells, we stably overexpressed SOX2 in T-5H-O cells using lentiviral particles for the expression of SOX2 cDNA (Figure 3A). SOX2 overexpression did not show any impact in the ability to form colonies in soft agar (Figure 3B,C) nor in the capacity to grow as tumorspheres (Figure 3D,E). Nevertheless, cells overexpressing SOX2 were more tumorigenic and grew tumors in immunodeficient mice significantly faster than controls cells (Figure 3F,G). Therefore, basal levels of SOX2 seems to be sufficient to efficiently promote clonal growth in vitro, however, certain microenviromental conditions present in the in vivo experiments might promote a long-term tumorigenic potential of those cells with higher expression of SOX2.

Taken together, the data from depletion and overexpression experiments suggest that SOX2 expression plays an active role in the initiation and progression of sarcomas thereby emerging as a biologically and clinically relevant feature.

### 2.3. SOX2 Activity Marks a Subpopulation of CSCs in Sarcoma

To assess whether cells expressing pluripotency factors like SOX2 behave as a CSC subpopulation with increased tumor-promoting ability, we made use of a lentiviral-based reporter system in which a composite SOX2/OCT4 response element (SORE6) coupled to a minimal cytomegalovirus (CMV) promoter controls the expression of GFP fluorescent reporter gene.

**Figure 4:**
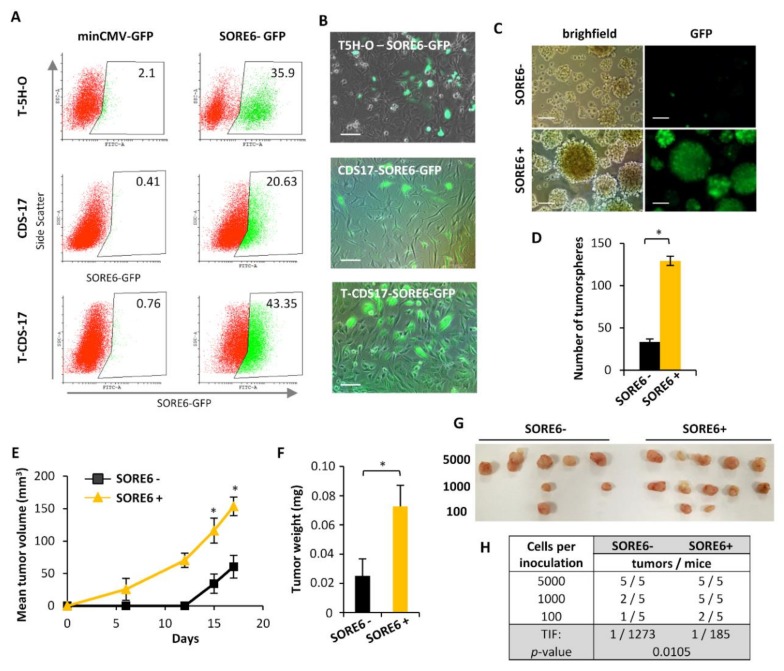
SORE6 activity marks a subpopulation of CSCs in sarcoma. (**A**) Flow-cytometry analysis showing the level of SORE6+ cells in T-5H-O-SORE6-GFP, CDS17-SORE6-GFP and T-CDS17-SORE6-GFP cultures or its corresponding minCMV-GFP control cells. (**B**) Fluorescence microscopy images showing cell subpopulations presenting GFP-associated SORE6 activity. Scale bars = 50 μm (**C**,**D**) Tumorsphere formation assay performed on SORE6^+^ and SORE6^−^cells. Representative images (**C**) and the quantification of number of tumorspheres generated in three independent experiments (**D**) are shown. Scale bars = 200 μm (**E**,**F**) In vivo tumor formation ability of SORE6+ and SORE6- subpopulations of T-5H-O-SORE6-GFP cells. (**E**) Tumor growth kinetics observed after inoculation of immunodeficient mice with 1 × 10^4^ cells of each population (n = 8 mice per series). (**F**) Average tumor weight at the end of the experiment. (**G**,**H**) In vivo limit dilution assay (LDA) to evaluate the tumor forming potential of SORE6+ and SORE6- T-5H-O-SORE6-GFP cells. (**G**) Images of the tumors formed in both series upon the inoculation of 5000, 1000 or 100 cells. (**H**) Quantification of the frequency of tumor initiating (TIF) cells using the ELDA software. The number of mice that grew tumors after 4 weeks and total number of inoculated mice for each condition is indicated. Error bars represent the standard deviation and asterisks indicate statistically significant differences (*: *p* < 0.05 by two-sided Student *t* test).

The inclusion of proteasome-targeting degron sequences in the reporter genes resulted in greater selectivity and temporal resolution [29]. This system allowed us to detect, monitor and isolate viable cells expressing transcriptionally active SOX2 and/or OCT4 by flow cytometry (Figure 4A) or live cell time-lapse microscopy (Figure 4B). Thus, we used these lentiviral construction to transduce T-5H-O cells, a patient-derived chrondrosarcoma primary cell line, CDS-17, and a cell line derived from a xenograft generated by CDS-17 cells, T-CDS-17, in order to generate lines with stable expression of the SORE6 construct (T-5H-O-SORE6-GFP, CDS-17-SORE6-GFP and T-CDS-17-SORE6-GFP) or its corresponding control without the SORE6 response element (T-5H-O-minCMV-GFP, CDS-17-minCMV-GFP and T-CDS-17-minCMV-GFP) which have been used as gating controls in the flow cytometry analyses. First, we found that the T-5H-O-SORE6-GFP, CDS-17-SORE6-GFP and T-CDS-17-SORE6-GFP cells displayed percentages of SORE6+ ranging between 20 and 40% (Figure 4A,B). Therefore, we use SORE6 activity to isolate SORE6+ and SORE6- subpopulations by flow cytometry in the three cell lines (Figure S4A–C). As expected, SORE6+ T-5H-O cells showed a significantly higher expression of SOX2 than the SORE6- subpopulation (Figure S4D,E). We also found that SORE6+ cells showed a much higher ability to form tumorspheres than SORE6- cells both in T-5H-O UPS cells (Figure 4C,D) and in CDS-17 and T-CDS17 chondrosarcoma cells (Figure S5). To study whether SORE6+ subpopulation was enriched in tumor-promoting cells, we inoculated 1 × 10^4^ cells of both T-5H-O SORE6- and SORE6+ subpopulations in immunodeficient mice and measured tumor formation over time. We observed tumor growth in the SORE6+ series as early as day 6 post-inoculation. On the other hand, SORE6- cells did not generate measurable tumor growth till day 15 after the inoculation and showed significant statistical differences in tumor volume with the SORE6+ series (Figure 4E). At the end-point, tumor weights confirmed that SORE6+ cells generated significantly larger tumors than those obtained from SORE6- cells (Figure 4F). To confirm and quantify the enrichment of the SORE6+ subpopulation in CSCs, we performed LDA comparing the ability of SORE6- and SORE6+ cells to initiate tumor growth in vivo. We found that SORE6+ cells produced tumors in all cases after the inoculation of 5000 or 1000 cells and in 2 out of 5 tumors (2/5) after the inoculation of 100 cells. On the other hand, SORE6- generated 5/5, 2/5 and 1/5 tumors after the injection of 5000, 1000 or 100 cells respectively (Figure 4G). Therefore, the tumor-initiation frequency (TIF) calculated using ELDA software was 7-fold higher in SORE6+ cells (1 tumor-initiating cell out of 185) compared to SORE6- cells (1 out of 1273) (Figure 4H).

These experiments suggest that high SOX2/OCT4 transcriptional activity, measured by SORE6 activity, could be used as a surrogate marker for CSCs in sarcomas.

### 2.4. SORE6 Response Element is a Valuable Tool to Monitor CSCs Response to Anti-tumor Treatments

Given its role as CSC marker, we aimed to test whether SORE6 activity could be useful to evaluate the effectivity of anti-tumor drugs to target CSCs subpopulations. Therefore, we studied the impact on the SORE6+ subpopulation of drugs used in the treatment of sarcomas, like doxorubicin, trabectedin and paclitaxel, as well as the mithramycin analog EC-8042 which has proven as highly efficacious in targeting CSCs in sarcoma [36].

**Figure 5:**
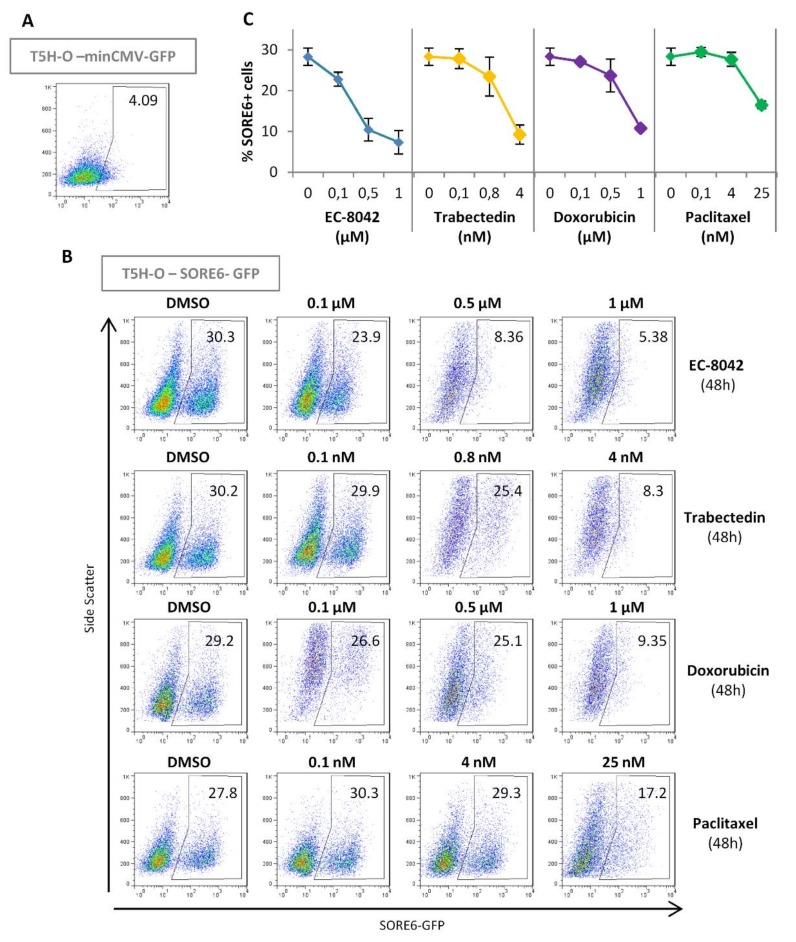
Dose-response effect of anti-tumor drugs on SORE6 positive CSCs. (**A**,**B**) Representative flow cytometry analysis of SORE6+ population in untreated T5H-O-minCMV-GFP (gating control) (**A**) or in T5H-O-SORE6-GFP cells treated with the indicated concentrations of EC-8042, trabectedin, doxorubicin or paclitaxel for 48 h (**B**). (**C**) Graph showing the mean ± standard deviation of three independent experiments.

**Figure 6:**
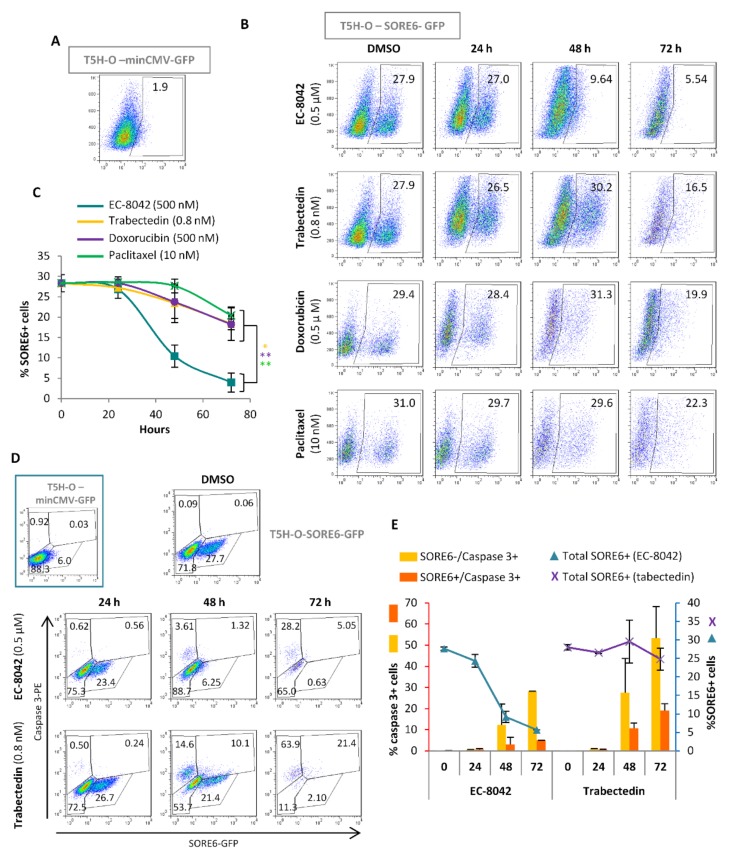
Differential effects of anti-tumor drugs on SORE6 positive cells. (**A**–**C**) Time course analysis of SORE+ cells during drug treatment. (**A**,**B**) Representative flow cytometry analysis of the SORE6+ population in untreated T5H-O-minCMV-GFP (gating control) (**A**) or in T5H-O-SORE6-GFP cells treated for with 0.5 µM EC-8042, 0.8 nM trabectedin, 0.5 µM doxorubicin or 10 nM paclitaxel for the indicated times (**B**). (**C**) Graph showing the mean ± standard deviation of three independent experiments. Asterisks indicate statistically significant differences with the EC-8042 series at 72 h (*: *p* < 0.05, **: *p* < 0.005; two-sided Student *t* test). (**D**,**E**) Biparametric flow cytometry analysis of SORE-GFP and active-caspase 3 levels. (**D**) Representative analysis in untreated T5H-O-minCMV-GFP (gating control, inset) or in T5H-O-SORE6-GFP cells treated with Trabectedin (0.8 nM) or EC-8042 (0.5 µM) for the indicated times. (**E**) Graphic representation of three independent experiments (mean ± standard deviation).

The IC_50_ values of these drugs in T-5H-O-SORE6-GFP cells were 307, 0.66, 7, and 288 nM for doxorubicin, trabectedin, paclitaxel and EC-8042 respectively (Figure S6). According to these values we evaluated SORE6 activity in T-5H-O cells in dose-response experiments using concentrations of each drug that induced low, medium (≈IC_60_), and high toxicity after 48 h of treatment. In these experiments, EC-8042 was the most efficient drug to reduce the SORE6+ subpopulation, being able to induce a 75% decrease of SORE6+ cells with a concentration in the order of its IC_60_. On the other hand, the rest of drugs only induced a clear regression of the SORE6 subpopulation when the higher concentrations were used (Figure 5). In addition, time course analysis after the treatment with concentrations in the order of the IC_60_ values also confirmed the higher potential of EC-8042 to eradicate SORE6+ cells in comparison with doxorubicin, trabectedin and paclitaxel (Figure 6A–C). This strong ability of EC-8042 to target SORE6+ cells was also evident in dose-response and time-course analysis performed in CDS-17-SORE6-GFP and T-CDS17-SORE6-GFP cells (Figure S7).

To better characterize the mechanism associated with the differential ability of these drugs to decrease SORE6+ cells, we simultaneously analyzed SORE6 activity and caspase-3 activation by flow cytometry in T-5H-O-SORE6-GFP cells treated with trabectedin or EC-8042. In these analyses we found that trabectedin was an efficient inductor of apoptosis both in SORE6+ and SORE6- cells. In addition, we found that EC-8042-treatment sharply reduced the percentage of SORE6+ cells even before the apoptotic effect become evident (Figure 6D,E). These results suggest that both drugs were able to target CSCs subpopulations using different mechanisms. On one hand, trabectedin eliminated SORE6+ cells through the induction of apoptosis and, on the other hand, EC-8042 would be able to switch-off SORE6-related transcriptional activity, thus possibly affecting their CSC-associated properties, prior to the induction of apoptosis.

**Figure 7:**
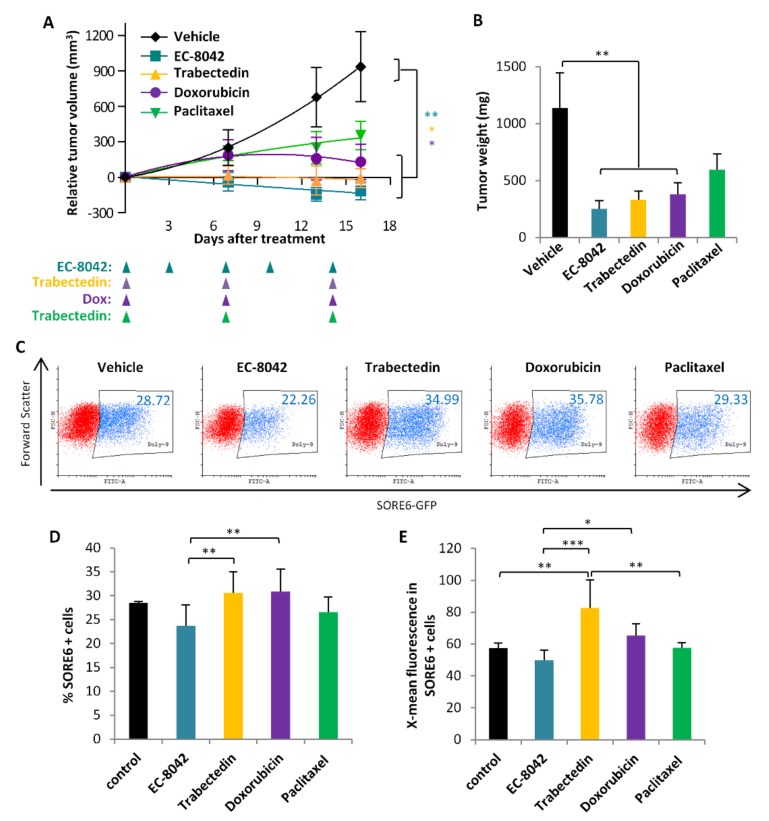
Evaluation of SORE6 activity after in vivo treatments. Mice with tumor xenografts established after the s.c. inoculation of 3 × 10^6^ T-5H-O-SORE6-GFP cells were randomly assigned to 5 different groups (*n* = 5 per group) and treated i.v. with saline buffer (control), EC-8042 (18 mg/Kg; every 3/4 days up to 5 doses), trabectedin (0.15 mg/Kg; every 7 days up to 3 doses), doxorubicin (4 mg/Kg; every 7 days up to 3 doses) or paclitaxel (20 mg/Kg; every 7 days up to 3 doses). (**A**) Curves representing the mean relative tumor volume (± SEM) of xenografts during the treatments. The timing of treatments is indicated, all mice received the last treatment on day 14 and tumors were extracted and analyzed on day 16. (**B**) Mean tumor weight (±SEM) at the end of the experiment. Asterisks indicate significantly different kinetics than the control series (*: *p* < 0.05, **: *p* < 0.005; one-way ANOVA). (**C**,**D**) Flow cytometry analysis of SORE6 activity in tumors collected and disaggregated 48 h after the last dose of the indicated treatments. Representative flow cytometry dot plots (**C**) and summary graphs representing the percentage of SORE6+ cells (mean ± standard deviation) (**D**) and the mean SORE-GFP fluorescence intensity (mean ± standard deviation) (**E**) are shown. Asterisks indicate statistically significant differences between series (*: *p* < 0.05, **: *p* < 0.005, ***: *p* < 0.0005; one-way ANOVA).

Next, we treated mice bearing T-5H-O-SORE6-GFP tumors with different drugs, using previously established treatment regimens [36,37,38], to evaluate their effect on SORE6+ cells in vivo. With the exception of paclitaxel, all drugs were able to significantly reduced tumor growth, being EC-8042 the most efficient treatment (Figure 7A,B). At the experimental end-point SORE6 activity was analyzed by flow cytometry in dissociated tumor cells. We found that EC-8042 was the only drug able to reduce the percentage and the fluorescence intensity of SORE6+ cells (Figure 7C–E). On the other hand, trabectedin or doxorubicin treatments produced a slight increase in the percentage of SORE6+ cells and fluorescence intensity, resulting in significant differences with the levels detected in EC-8042-treated tumors (Figure 7C–E).

Altogether, these results prove the usefulness of analyzing SORE6+ activity to evaluate the activity of anti-tumor drugs to target CSCs in sarcoma both in vitro and in vivo.

## 3. Discussion

Similar to hematological malignancies and other solid tumors, intra-tumor heterogeneity in sarcomas may be explained, at least in part, by the emergence of subpopulations of CSCs which guide tumor growth and dissemination. The stemness state in sarcomas is orchestrated by the expression of pluripotency factors such as OCT3/4, NANOG, KLF4, and SOX2 [12,13,16,18]. Among them, SOX2 has been shown as a common CSC-related factor in different types of sarcoma [18,39]. The pro-tumorigenic role of SOX2 has been particularly well described in osteosarcoma models. Knockdown of this factor in osteosarcoma cell lines or in the osteoblastic lineage of an osteosarcoma mouse model resulted in the loss of proliferative potential in vitro and a drastic reduction of tumor formation in vivo [19,20,21]. Besides osteosarcoma, clues for pro-stemness and/or pro-tumorigenic role for SOX2 has also been reported in Ewing sarcoma [40,41] and embryonal rhabdomyosarcoma [42]. In addition, the level of SOX2 expression in a panel of primary sarcoma cell lines have been positively correlated with the ability to grow tumors in immunodeficient mice [17]. In line with these previous works, our knockdown experiments further expand the findings regarding the key role of SOX2 in sustaining tumorigenicity to a model of UPS. In addition, we show that SOX2 overexpression, explored for the first time in sarcoma, also support the prominent role of SOX2 in sarcomagenesis.

We found that SOX2 is expressed in 28% of an array of 88 sarcoma patients, being UPS, synovial sarcomas and Ewing sarcomas those presenting a higher percentage of positive cases in concordance with the results of previous reports [43,44]. In our series, SOX2 expression significantly correlated with tumor grade, poor differentiation, invasive potential and poor patient survival. Similar results have been recently reported for Ewing Sarcoma [43], thus reinforcing their key role in sarcoma development and disease progression. The clinical significance of OCT4 expression has been barely addressed in sarcomas. In our series of patient samples, a weak expression of OCT4 was only detected in a small subset of sarcoma samples (11%), being synovial sarcoma the subtype with a higher percentage of positive samples (56%). Even though OCT4 expression was not significantly correlated with any clinicopathologic parameter and showed no impact on patient survival in our cohort of sarcoma patients, we cannot discard that the analysis of larger series of patients could unravel a clinically relevant role for OCT4 in specific sarcoma subtypes.

Given the relevant role of SOX2 in tumorigenicity, here we used the SORE6 system [29] to study whether those subpopulations showing SOX2/OCT4 transcriptional activity behave as bona-fide CSCs with higher tumor-initiating potential than other subpopulations. SOX2-based reporter systems were previously used to demonstrate the CSC phenotype of SOX2-expressing subpopulations in glioma, breast, prostate, bladder or head and neck cancers [4,8,24,26,27,28,29,30,31], although this strategy remained unexplored in sarcomas. In addition, a plasmid containing the human OCT4 promoter driving the expression of GFP was used to show that OCT4-expressing osteosarcoma cells were much more tumorigenic than OCT4 negative cells [22]. In line with these works, we found that SORE6+ UPS cells displayed greater potential than SORE6- cells to form tumorspheres in vitro and to develop tumors in vivo, thus confirming their CSC phenotype. In addition, we also detected a 20% of SORE6+ cells in a low passaged patient-derived chondrosarcoma cell line (CDS-17). Interestingly, this percentage was increased to 40% upon growth of CDS17 cells in a immunodeficient mice (xenograft TCDS-17 line). Considering that T-CDS17 displayed increased aggressiveness (higher invasion and tumor formation ability) than CDS-17 cells [45], the increase in SORE6+ cells could respond to an increase of the CSC burden during tumor progression and adaptation to new microenvironments. Similar findings regarding the gain of aggressiveness upon in vivo tumor growth associated to an increase of CSC markers, such as ALDH activity or OCT4 expression, have also been described in different types of sarcoma [22,35,45,46]. These findings support that serial transplantation could represent an efficient way of enriching/selecting CSC subpopulations [18].

In previous studies, we have reported that drugs already approved for sarcoma treatment such as trabectedin and experimental compounds such the mythramycin analog EC-8042 were able to target CSC subpopulations (tumorsphere cultures and/or Aldefluor-positive cells) in sarcomas with a higher efficacy than doxorubicin [36,38]. Here we used the SORE6 system to analyze both in vitro and in vivo models the effectiveness of these drugs and other chemotherapeutics used to treat sarcomas such as doxorubicin and paclitaxel to target CSCs. EC-8042 was the most efficient drug to target SORE6+ cells in vitro. Noteworthy, the reduction of SORE6+ subpopulations after in vivo treatment with EC8042 was not so efficient as observed in vitro. We may speculate that this difference could be due to the pharmacokinetic behavior of drugs in cells and animal models and/or the influence of factors from the tumor microenvironment. Nevertheless, EC8042 was the only treatment able to reduce this subpopulation in vivo. After EC8042 treatment, SORE6+ cells disappeared before apoptosis become evident, thus suggesting that EC-8042 was able to repress the expression of SOX2, as we previously observed in a related myxoid liposarcoma model [36]. According to this, it was reported that mithramycin was able to reduce in vivo proliferation of glioblastoma cells through the downregulation of SOX2 expression and its target genes [47]. Likewise, mithramycin was able to abrogate tumor growth in medulloblastoma by targeting SOX2-expressing CSCs [48]. Given that EC-8042 is 10-fold less toxic than mithramycin [49], it could represent a suitable therapeutic option to eliminate CSCs in sarcomas.

Although trabectedin was not as selective as EC-8042 to eliminate SORE6+ cells, this drug proved to be an efficient apoptotic inductor in both SORE6- and SORE6+ subpopulations, similar to previous findings demonstrating its ability to eliminate tumorsphere and Aldefluor-positive cells in the same sarcoma model [38]. Therefore, our work show that different drugs may target CSCs in sarcoma using different mechanisms and also that the SORE6 system is a valuable tool to dynamically evaluate the activity of anti-tumor drugs to target CSCs in sarcoma as seen in other tumor types [29,30].

## 4. Materials and Methods

### 4.1. Cell Culture, Drugs and Ethics Statement

The UPS cell line T-5H-O and the chondrosarcoma cell lines CDS-17 and T-CDS17 were previously characterized (Supplementary Information) [32,33,34,35,45]. Tumorsphere formation and soft agar colony formation assays were performed as previously described [35,36]. Cell suspensions were counted in a haemocytometer using tryplan blue staining to discard non-viable cells both for in vitro and in vivo experiments. The percentage of viable cells in all conditions was always higher than 95%. Trabectedin (PharmaMar, Madrid, Spain), paclitaxel (Selleckchem, Houston, TX, USA), doxorubicin (Sigma, St Louis, MO, USA) and EC-8042 (EntreChem, Oviedo, Spain) were prepared as described in Supplementary Information. All experimental protocols have been performed in accordance with institutional review board guidelines and were approved by the Institutional Ethics Committee of the Principado de Asturias (ref. 45/16). All samples and data from human origin were provided by the Principado de Asturias BioBank (PT17/0015/0023) after obtaining signed informed consent.

### 4.2. Lentiviral Constructions and Cell Transduction

Conditional depletion of SOX2 was achieved using a doxycycline-inducible system obtained from Addgene (Cambridge, MA, USA) (Tet-pLKO-puro-SOX2; plasmid 47540) [50]. In addition, we also used a PLKO.1 lentivirus shRNA vector targeted against SOX2 (TRCN0000085748) together with the corresponding empty shRNA vector as a negative control (Dharmacon Lafayette, CO, USA). As an alternative method to knockdown SOX2 expression we used a specific siRNA (Supplementary Information). The lentiviral constructions to overexpress SOX2 (pSin-EF2-SOX2-Pur; addgene plasmid 16577) or GFP (used as a control) (pSin-EF2-GFP-Pur) cDNAs were kindly donated by Maria dM. Vivanco (CIC bioGUNE, Derio, Spain). Lentiviral reporter systems in which a composite SOX2/OCT4 response element (SORE6) coupled to a minimal cytomegalovirus (mCMV) drive the expression of GFP (SORE6-mCMVp-dsCopGFP-Puro) and its corresponding control lacking SORE6 (mCMVp-dsCopGFP-Puro) were previously generated and characterized [29] and were kindly donated by Dr. L.M. Wakefield (National Cancer Institute, Bethesda, MD, USA). Generation of lentiviral particles were performed as previously described [34]. Transduced cells were positively selected through a treatment with puromycin 30 mg/mL for 6 days. To achieve an inducible expression of SOX2 shRNA in vitro, cells were treated with 2 μg/mL of doxycycline (Sigma).

### 4.3. Flow Cytometry and Cell Sorting

The level of SORE6-driven GFP fluorescence in untreated cultures or after different drug treatments were analyzed and/or SORE6+ and SORE6- subpopulations were sorted by flow cytometry using a BD FACS Aria II Cell Sorter (BD Bioscience, Erembodegem, Belgium). Cells transduced with the minCMVp-GFP lentivirus were used as matched SORE6 negative control for gating purposes. In these analyses, dead cells were excluded by propidium iodide (0.5 μg/mL) staining (Figure S8). To analyze the induction of apoptosis in SORE6+ and SORE6- subpopulations, unfixed cells were assayed for active caspase-3 immediately after treatment using the PE Active Caspase-3 Apoptosis Kit (BD Bioscience) according to the manufacturer’s instructions and the level of GFP (SORE6) and PE (Caspase 3) fluorescence was simultaneously detected by flow cytometry. SOX2 and OCT4 expression was detected by flow cytometry in 70% ethanol-fixed cells using an anti-SOX2 antibody from Thermo Fisher (Waltham, MA) (PA1-094); 1: 1000 dilution) or anti-OCT4 antibody from AbCam (Cambridge, UK) ((ab19857); 1: 1000 dilution).

### 4.4. Western Blotting

Whole cell protein extraction and Western blot analysis were performed as previously described [36]. Antibodies used are described in Supplementary Information. Uncropped images of the Western Blottings are shown as in Figure S9.

### 4.5. RT-qPCR Assays

The expression of SOX2 was assessed by qPCR as described in Supplementary Information.

### 4.6. In Vivo Tumor Growth

Female NOD/SCID mice of 6-7 weeks old (Janvier Labs, St Berthevin, France) were inoculated subcutaneously (s.c.) as described [36]. In experiments aimed to evaluate the effect of anti-tumor drugs, mice with tumor xenografts with a volume of approximately 300 mm^3^ were randomly assigned to receive the following intravenous treatments: vehicle (saline, every 7 days up to 3 doses), EC-8042 (18 mg/Kg; every 3/4 days up to 5 doses), trabectedin (0.15 mg/Kg; every 7 days up to 3 doses), doxorubicin (4 mg/Kg; every 7 days up to 3 doses) or paclitaxel (20 mg/Kg; every 7 days up to 3 doses). Treatment schedules were optimized according to the therapeutic window of the different drugs [36,37,38]. To analyze the effect of the conditional knockdown of SOX2, mice inoculated with cells expressing or not the Tet-pLKO-puro-SOX2 lentiviral vector received a daily intraperitoneal dose of doxycycline (50 mg/kg). Tumor size was measured with a caliper 2–3 times a week and tumor volume was determined using the equation (D × d2)/6 × 3.14, where D is the maximum diameter, and d is the minimum diameter. Relative tumor volume (RTV) for every xenograft was calculated as follows: RTV = tumor volume at day of measurement (V_t_) − tumor volume at the beginning of the treatment (V_0_). Tumor volumes, or RTV in drug-treated experiments, for all mice in each group were averaged to obtain the mean tumor volume for the corresponding group. Animals were sacrificed by CO_2_ asphyxiation and tumors were weighted. To determine the effect of drugs on SORE6 activity, tumors were dissociated into single cell suspensions using MACS Tissue Dissociation Kit and the GentleMACS Dissociator system (Miltenyi Biotec, Bergisch Gladbach, Germany) and the SORE6-positive subpopulations were quantified by flow cytometry. In limited dilution assays (LDA) animals were sacrificed 4 weeks after cells inoculation. In these experiments, relative tumor-initiating frequency (TIF) was calculated using the ELDA software. All experimental protocols were carried out in accordance with the institutional guidelines of the University of Oviedo and were approved by the Animal Research Ethical Committee of the University of Oviedo prior to the study (Ref. PROAE11/2014).

### 4.7. Patients and Immunohistochemical Analysis

Paraffin-embedded tissues from 90 patients with sarcoma who underwent resection of their tumors at the Hospital Universitario Central de Asturias (HUCA) were used in this study. Tumor grade was evaluated in H&E-stained preparations using the French Federation of Comprehensive Cancer Centers grading system (Supplementary Information). Tissue microarray was constructed as previously described [51]. Immunohistochemical analysis of SOX2 and OCT4 expression was performed as detailed in Supplementary Information. The immunostaining was scored blinded to clinical data by two independent observers as negative or positive nuclear staining (> 1% positive nuclei).

### 4.8. Statistical Analysis

For the in vitro experiments and the tumor growth experiment in vivo, the statistical analysis was performed using the GraphPad Prism software (GraphPad Software, Inc, La Jolla, CA, USA). All data are represented as mean (±SD or SEM as indicated) of at least three independent experiments unless otherwise stated. Student’s *t* test was performed to determine the statistical significance between groups. Multiple comparisons of the data were performed using the one-way ANOVA. For immunohistochemical analysis, the experimental results distributed among the different clinical groups of tumors were tested for significance employing the χ2 test (with Yates’ correction, when appropriate). Survival curves were calculated using the Kaplan-Meier product limit estimate. Differences between survival times were analyzed by the log-rank method and the Hazard Ratio was calculated by univariate Cox regression analysis. All statistical analysis was carried out with the software package SPSS 24 (SPSS, IBM corp). All tests were two-sided and *p* < 0.05 values were considered statistically significant.

## 5. Conclusions

Overall, our results indicate that SOX2 is a critical stemness factor able to increase the tumorigenic properties of sarcoma cells. Notably, SOX2 expression correlate with advanced-disease related parameters in patients, therefore, suggesting its possible usefulness as prognostic marker in sarcoma. Moreover, the transcriptional activity of SOX2 and/or OCT4, measured using the SORE6 reporter, is a bona fide CSC marker in sarcoma and constitutes an excellent approach for testing the effectiveness of anti-tumor treatments to target CSCs.

## Supplementary Materials

The following are available online at https://www.mdpi.com/2072-6694/12/4/964/s1, Supplementary Materials and Methods, Figure S1: Immunohistochemical analysis of OCT4 expression in sarcoma patients and associations clinical data, Figure S2: OCT4 expression in SOX2-depleted cells, Figure S3: SOX2 depletion inhibited colony formation in soft agar and tumorsphere growth, Figure S4: Flow cytometry sorting of SORE6 positive and negative subpopulations, Figure S5: Tumorsphere growth ability of SORE6+ and SORE6- subpopulations in CDS17 and T-CDS17 cell lines, Figure S6: Dose-response toxicity curves, Figure S7: Effect of EC-8042 on the SORE6+ subpopulation of chondrosarcoma patient-derived cell lines, Figure S8: Gating strategy used to analyze SORE6 activity, Figure S9, Whole Western blotting images.
